# Targeting B cell malignancies through human B cell receptor specific CD4 T cells

**DOI:** 10.1186/2051-1426-3-S2-P59

**Published:** 2015-11-04

**Authors:** Jinsheng Weng, Flavio Egidio Baio, Kelsey Moriarty, Hiroki Torikai, Hua Wang, Zhiqiang Liu, Sourindra Maiti, Dongho Gwak, Michael Popescu, Soung-chul Cha, Sattva S Neelapu, Larry Kwak

**Affiliations:** 1 The University of Texas MD Anderson Cancer Center, Houston, TX, USA

## Background

The B cell receptor (BCR) expressed by a clonal B cell tumor is a tumor specific antigen (idiotype). However, the T cell epitopes within human BCRs which stimulate protective immunity still lack detailed characterization.

## Methods

In this study, we identified 17 potential CD4 T cell peptide epitopes derived from BCR heavy and light chain variable region sequences. Detailed analysis revealed these CD4 T cell epitopes stimulated Th1 CD4 T cells to directly recognize the autologous tumors by secretion of IFN-γ, indicating the epitopes are processed and presented by tumors.

## Results

One BCR peptide-specific CD4 T cell line was also cytotoxic and lysed autologous tumor cells through the perforin pathway. Sequence analysis of the epitopes revealed 10 were potentially shared by multiple primary patients' tumors, and 16 had the capacity to bind more than one HLA DRB1 allele. T cells stimulated by shared epitopes recognized primary tumors expressing the same sequences on different HLA DRB1 alleles.

## Conclusions

In conclusion, we identified multiple BCR-derived CD4 T cell epitopes with promiscuous HLA binding that are shared by up to 36% of patients, suggesting a strategy to overcome the requirement for individual preparation of therapeutic agents targeting idiotype.

